# Effect of anaerobic phases length on denitrifying dephosphatation biocenosis – a case study of IFAS-MBSBBR

**DOI:** 10.1186/s12866-020-01896-3

**Published:** 2020-07-24

**Authors:** Anna Gnida, Monika Żubrowska-Sudoł, Katarzyna Sytek-Szmeichel, Jolanta Podedworna, Joanna Surmacz-Górska, Dorota Marciocha

**Affiliations:** 1 Department of Environmental Biotechnology, Faculty of Energy and Environmental Engineering, Silesian University of Technology, 2A Akademicka St., 44-100 Gliwice, Poland; 2 Faculty of Building Services, Hydro and Environmental Engineering, Warsaw University of Technology, Nowowiejska Str. 20, 00-653 Warsaw, Poland

**Keywords:** Denitrifying dephosphatation, Polyphosphate accumulating organisms, Moving bed reactor, Activated sludge, Biofilm, Wastewater treatment

## Abstract

**Background:**

The study aimed to evaluate the influence of the duration times of anaerobic phases on the bacterial biocenosis characterisation while denitrifying dephosphatation in the Integrated Fixed-Film Activated Sludge – Moving-Bed Sequencing Batch Biofilm Reactor (IFAS-MBSBBR). The experiment was conducted in a laboratory model. The study consisted of four series, which differed in terms of the ratio of the anaerobic phases.

duration concerning the overall reaction time in the cycle. The anaerobic phases covered from 18 to 30% of the whole cycle duration. During the reactor performance that took 9 months, the influent and effluent were monitored by analysis of COD, TKN, NH_4_-N, NO_2_-N, NO_3_-N, TP, PO_4_-P, pH, alkalinity and the phosphorus uptake batch tests. Characterisation of the activated sludge and the biofilm biocenosis was based on fluorescent in situ hybridisation (identification of PAO and GAO) and the denaturing gradient gel electrophoresis patterns.

**Results:**

The organic compounds removal was high (more than 95.7%) independently of cycle configuration. The best efficiency for nitrogen (91.1%) and phosphorus (98.8%) removal was achieved for the 30% share of the anaerobic phases in the reaction time. Denitrifying PAO (DPAO) covered more than 90% of PAO in the biofilm and usually around 70% of PAO in the activated sludge. A substantial part of the polyphosphate accumulating organisms (PAO) community were *Actinobacteria*. The denitrifying dephosphatation activity was performed mainly by *Accumulibacter phosphatis*.

**Conclusions:**

High nutrient removal efficiencies may be obtained in IFAS-MBSBBR using the denitrifying dephosphatation process. It was found that the length of anaerobic phases influenced denitrification and the biological phosphorus removal. The extension of the anaerobic phases duration time in the reaction time caused an increase in the percentage share of denitrifying PAO (DPAO) in PAO. The biocenosis of the biofilm and the activated sludge reveal different species patterns and domination of the EBPR community.

## Background

Efficient removal of nutrient compounds such as carbon, nitrogen, and phosphorus from different types of wastewater is still one of the priority issues concerning the environment and its protection. Many wastewater treatment plants (WWTPs) suffer from a too low load of organic compounds in wastewater concerning the requirements associated with the removal of nitrogen and phosphorus [1]. The problem is usually solved by a dosage of an external carbon source or by performing chemical dephosphatation, which increases the operating costs of the plant. The alternative seems to be the use of denitrifying dephosphatation, which is based on the obtainment of a specific group of organisms capable of accumulating orthophosphates in anoxic conditions with a simultaneous reduction of nitrates to nitrogen gas.

The discovery of a bacterial group capable of binding the excess of phosphates under anoxic conditions and at the same time reducing nitrate or nitrite to nitrogen gas (DPAO, denitrifying polyphosphate accumulating organisms) resulted in acknowledging the possibility of using this phenomenon (denitrifying dephosphatation) to a synergistic removal of nitrogen and phosphorus from wastewater with significantly lower demand for organic carbon. Denitrifying dephosphatation has been attracting the attention of researchers for more than two decades now. It shows important advantages apart from simultaneous dephosphatation and denitrification. These are a lower demand for organic carbon, reduction of aeration (lower oxygen demand), and decline in the biomass growth [2, 3]. Although the process allows reducing wastewater treatment costs, it is not ideal and easy to apply. Denitrifying dephosphatation shows a lower denitrification efficiency in comparison with traditional solutions for the N and P removal. Moreover, the demand for N:P of 7 is rarely encountered in municipal wastewater [3]. Thus, ensuring optimal conditions and the enrichment of DPAO can be difficult.

Due to the mentioned advantages and disadvantages of the process, it is necessary to search for such working conditions of the bioreactor in which the performance of denitrifying dephosphatation represents the highest possible share in the total efficiency of nitrogen and phosphorus removal represented by all processes. The factors that promote growth and stimulate the activity of DPAO in activated sludge and favour the denitrifying orthophosphate consumption have a significant influence on the efficiency increase in denitrifying dephosphatation. Enrichment of DPAOs requires conditions inhibiting the growth of other bacteria that can compete with them. DPAOs proliferate particularly easily in a continually repeating sequence of alternating anoxic and anaerobic conditions, while in anaerobic conditions sufficient quantities of a readily biodegradable organic substrate (e.g. acetates) must be available [4, 5]. The load of the substrate should provide an adequate efficiency of the orthophosphates release and an adequate level of the intracellular substrate available in a further zone with the presence of electron acceptors [6]. It has been shown that the rate of the phosphorus uptake and the denitrification rate increase along with increasing the initial levels of polyhydroxyalkanoates (PHA) in the DPAO’s cells [6]. However, obtaining a DPAO-rich community is subjected to an additional assurance - the lack of “portability” of acetates and nitrates to the anoxic and anaerobic zones, respectively. The whole organic substrate should be used in anaerobic conditions and not be available in anoxic ones, while all the nitrates should be used in anoxic conditions and not enter the anaerobic zone. The lack of organic compounds in anaerobic and nitrates in anoxic conditions restrains the growth of heterotrophic bacteria not accumulating PHA [7].

Measurable benefits that can be achieved using the process of denitrifying dephosphatation stimulate the search of technological systems which would ensure a synergistic removal of nitrogen and phosphorus compounds from wastewater. Different systems were tested for the enrichment of DPAO and an efficient C, N, and P removal. The researchers tested systems with separate biomass for nitrification and denitrifying dephosphatation (A_2_-conventional system [2] and A_2_-SBR [7, 8] or one-reactor systems (A2O [9], UCT [10])).

Denitrifying dephosphatation can also be achieved in the sequencing batch reactors. The examples of such solution are: SBR (Sequencing Batch Reactor) [11] with activated sludge, SBBR (Sequencing Batch Biofilm Reactor, [12], or pure MBSBBR (Moving Bed Sequencing Batch Biofilm Reactor)) [13, 14], in which biomass was developed mostly in a biofilm formed on the surface of moving carriers, and IFAS-MBSBBR (Integrated Fixed-Film Activated Sludge – Moving-Bed Sequencing Batch Biofilm Reactor) [15], in which biomass was developed as activated sludge and biofilm. In the case of IFAS-MBSBBR, after the anaerobic phase that ensures the removal of readily biodegradable organic compounds, an aerobic phase occurs, where the oxidation of ammonia is held. In such a reactor, oxygen conditions in the wastewater and on the surfaces of activated sludge or biofilm flocs may coexist, as well as anoxic conditions inside the flocs and biofilm. Nitrates and orthophosphates can penetrate between biomass layers and be constantly available to DPAO bacteria. The phenomenon was postulated among other things by Pastorelli et al. [13]. This paper presents the results of research on the influence of the duration of the anaerobic phases on the bacterial biocenosis performing the denitrifying dephosphatation in IFAS-MBSBBR. The hypothesis was that the elongation of anaerobic phases improves the enrichment of biomass in PAO representatives and let to obtain better removal efficiencies of nutrients.

## Results

### Reactor performance

During the whole study, changes in the characteristics of the influent and effluent were controlled, which allowed to assess the efficiency of the organic compounds removal, nitrification, denitrification, and biological phosphorus removal.

The data summarised in Table 1 demonstrate that regardless of the ratio of the duration of the anaerobic phases in relation to the overall reaction time in the cycle, high and comparable efficiency of the organic compound removal was obtained with an average value of 96.2 ± 0.9% in all the series. The COD in the effluent did not exceed 30 mg O_2_/l. Moreover, a highly effective process of the ammonia nitrogen oxidation in all the series was achieved. The monitored concentrations of nitrite and FNA were always under the toxic thresholds. It is worth stressing that along with an increase of the anaerobic phases duration time (a decrease of the aeration duration time) no decrease in the nitrification efficiency was observed. What is more, a higher nitrification process efficiency was achieved in series A4 (95.7 ± 1.6%) than in series A1 (92.6 ± 5.3%). When analysing the efficiency of the denitrification process, it can be noted that an increase in the duration time of the anaerobic phases did not influence the nitrogen removal. The highest efficiency of the denitrification process (91.1 ± 1.8%) was achieved in series A3, probably due to the introduction of multiple feeding, which allows for better use of the organic compounds by the biomass present in the reactor. Our previous studies [15] have shown that the number of feedings in the cycle has a significant impact on the effectiveness of the denitrification process in the IFAS-MBSBBR system.

**Table 1:** The efficiency of selected biological processes performed in MBSBBR in the study (average ± standard deviation (number of samples))

series	Organic compounds removal (COD), %	Nitrification efficiency, %	Denitrification efficiency, %	Biological phosphorus removal efficiency, %
**A1**	96.7 ± 0.9 (13)	92.6 ± 5.3 (13)	87.3 ± 4.6 (13)	68.1 ± 6.5 (13)
**A2**	95.7 ± 0.8 (14)	94.9 ± 2.3 (14)	88.5 ± 3.3 (14)	85.8 ± 14.4 (14)
**A3**	96.2 ± 1.0 (18)	92.6 ± 5.3 (18)	91.1 ± 1.8 (18)	98.8 ± 1.3 (18)
**A4**	96.3 ± 0.7 (15)	95.7 ± 1.6 (15)	85.4 ± 2.4 (15)	97.8 ± 3.2 (15)

According to the data presented in Table 1, it was also found that along with an increase in the duration of the anaerobic phases, an increase in the efficiency of the biological phosphorus removal was observed. The efficiency of the phosphorus removal in series A1 (anaerobic phases duration: 70 min /cycle) was only 68.1 ± 6.5%, and it increased to the level of 85.8 ± 14.4% in series A2 (100 min./cycle). The anaerobic phases in series A3 and A4 lasted 120 and 130 min./cycle, respectively, and contributed to an increase in the efficiency of removing phosphorus to more than 97.8%. The observed influence of the duration of the anaerobic phases on the biological phosphorus removal process resulted from a better gathering up of the storage substance by the PAOs under anaerobic conditions.

**Fig. 1:**
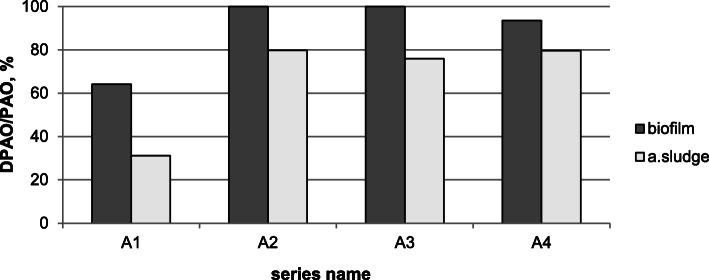
Percentage share of DPAO in PAO present in the activated sludge and the biofilm; dark grey – biofilm, light grey – activated sludge

The scope of the experiment also included the phosphorus uptake batch tests. As shown in Fig. 1, the extension of the anaerobic phases duration time in the cycle from 70 min. (series A1) to 100 min. (series A2) allows increasing the ratio of DPAO/PAO in the activated sludge as well as in the biofilm. The percentage share of DPAO in PAO increased from 31.2 and 64.1% to 79.8 and 100% in the activated sludge and the biofilm, respectively. This phenomenon resulted from the possibility of a higher uptake of readily biodegradable organic carbon under the anaerobic conditions.

Along with a further extension of the duration of the anaerobic phases, no significant differences in the percentage share of DPAO in PAO were observed. The obtained results also indicated that a significantly higher value of the percentage share of DPAO in the PAO population was always achieved in the biofilm. Such an observation suggests that the biofilm might be a more favourable biotope for the development of DPAO.

### PCR-DGGE

PCR-DGGE was used to assess the similarity of the microbial community of the activated sludge and the biofilm. At the end of each of the four series of the experiment, the composition of the total bacterial community was analysed with the 16S rRNA gene amplified with 338f-GC and 518 r primers.

**Fig. 2:**
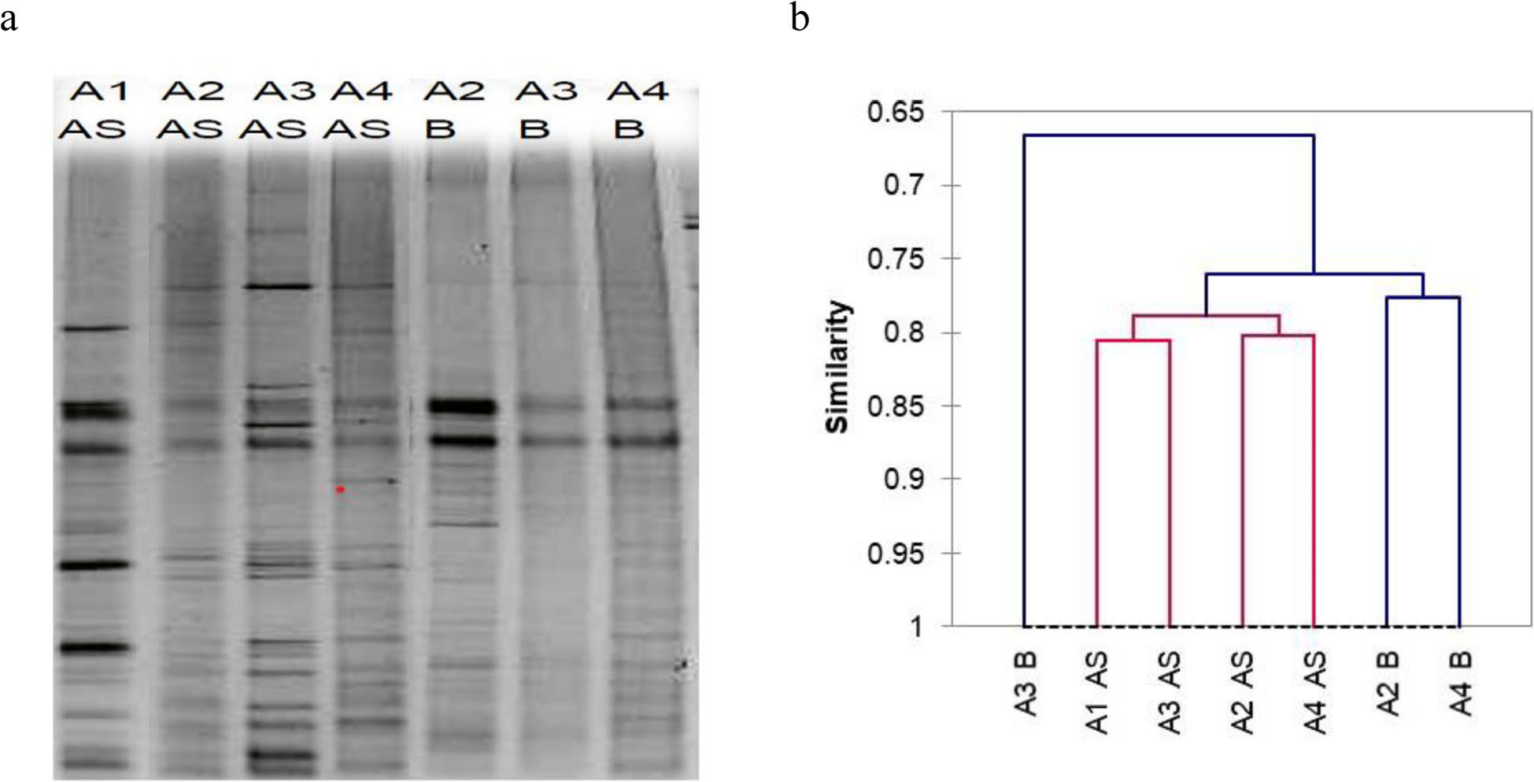
PCR-DGGE-based genotypic structure of the bacterial community during the study (**a**) and UPGMA clustering of DGGE bands by pattern similarity (**b**); AS - activated sludge; B - biofilm; A1 - A4 – series’ name (see: Table 3 for explanation); the gel image was inverted and cropped because the gel contained samples that are not discussed in this article

Figure 2a presents the genotypic structure of the activated sludge or the biofilm bacterial community (based on PCR-DGGE, 16S rRNA gene) in MBBSBR.

The clustering analysis results are presented in Fig. 2b. It has to be pointed out that the analysis uses binary data, which means that only the presence and position of the bands, and not their intensity, were taken into account.

It can be seen from Fig. 2b that the activated sludge samples belong to the same group (cluster), and all the biofilm samples belong to the other group. The highest similarity, according to UPGMA, was obtained between samples A2 and A4, both in activated sludge and biofilm.

### FISH

**Fig. 3:**
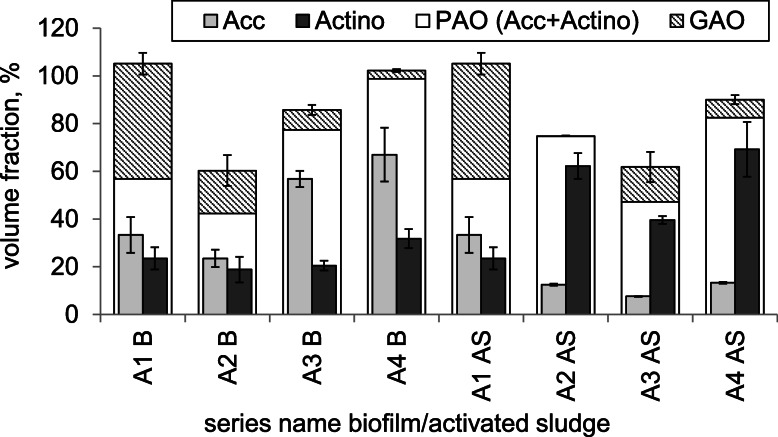
Percentage abundance of Candidatus “*Accumulibacter phosphatis*” (AccPAO; medium grey), Actinobacterial PAO (ActinoPAO; dark grey), all PAO (white,) and glycogen accumulating organisms (GAO; hatching) in the bacterial community from MBSBBR during the determination of the optimal duration of the anaerobic phase or the number of feedings (AS - activated sludge; B - biofilm; A1 - A4 – series’ name)

Candidatus “*Accumulibacter phosphatis*” (AccPAO) and Actinobacterial PAO (ActinoPAO) were found in each taken sample. Figure 3 shows the contribution of both groups in the bacterial community of the activated sludge or the biofilm sampled at the end of each series. The volume fraction of Candidatus “*Accumulibacter phosphatis*” was between 33 and 67% for the biofilm and 8–33% for the activated sludge (Fig. 3). The sum of PAO and GAO sometimes exceeds 100%. Such an overestimation may result from the lack of detection of some bacteria by the EUB probe while being detected by the specific probe [16], or rather from the image acquisition settings that are difficult to acquire and maintain identical for all samples and probes used [17].

**Fig. 4:**
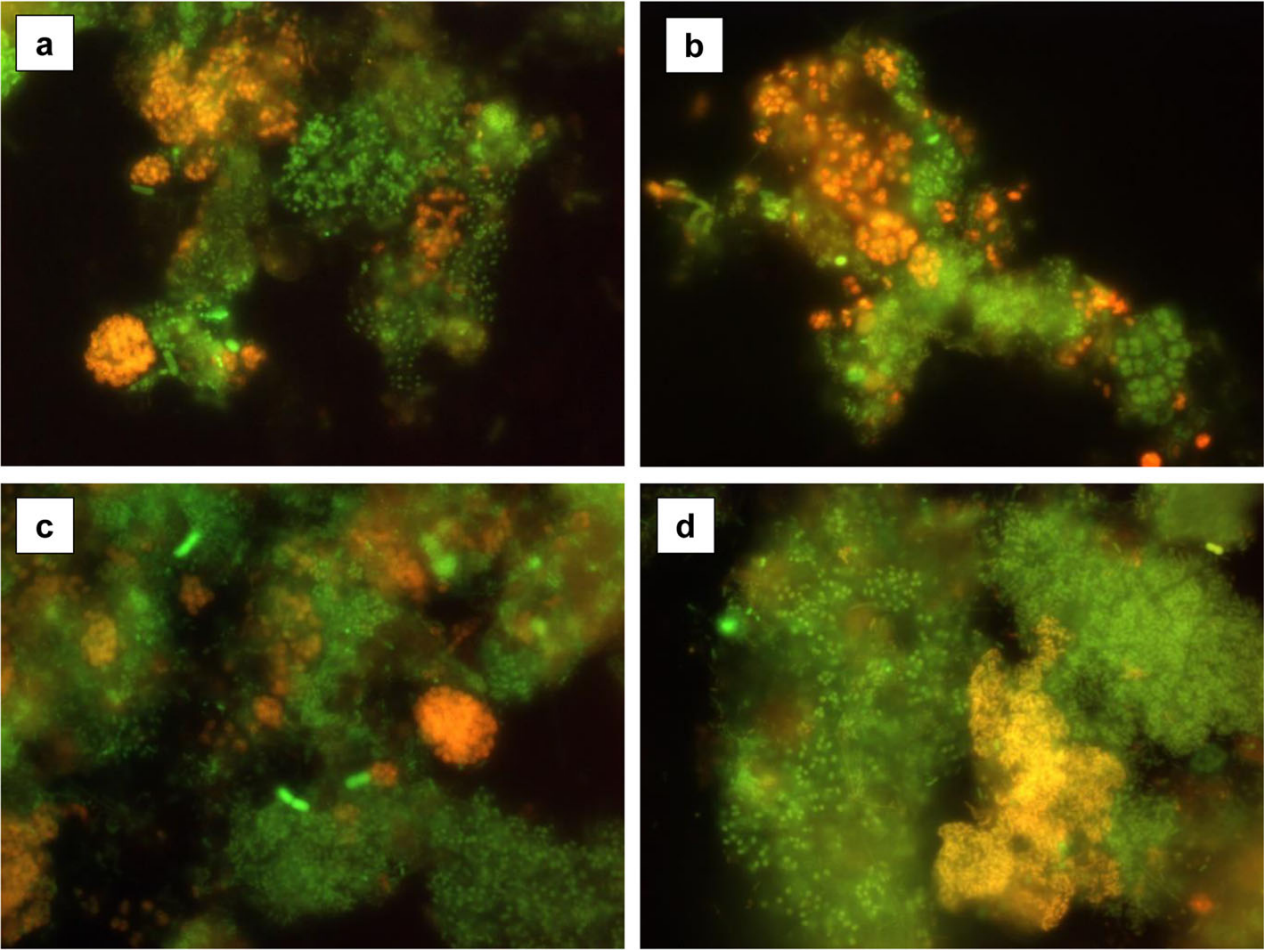
Detection of phosphate accumulating bacteria by fluorescence in situ hybridizations (FISH). Detection of PAO using a mixture of 16S rRNA gene specific probes PAO462, PAO651 and PAO846 in activated sludge from series A3 (**a**) and A4 (**b**). Detection of *Accumulibacter phosphatis* clade II using 16S rRNA gene specific probe Acc-II-444 in biofilm from series A4 (**c**) and Actinobacterial PAO using 16S rRNA gene specific probe Actino658 in biofilm from series A3 (**d**). In all cases, all bacteria were detected by a mixture of 16S rRNA gene probe EUB338, EUB338II and EUB338III. All bacteria appear green, while specific probes appear yellow/orange due to dual labelling with green and red. Images captured by Katarzyna Drzewiecka

In the biofilm, there was app. Twice as much AccPAO in the last two series (A3 B and A4 B) than in the first two, but in the activated sludge these values diminished with an increasing percentage of the anaerobic phases. ActinoPAO covered a narrower range in the biofilm 19–32%, but in the activated sludge its share almost tripled from 24% in series A1 AS to almost 70% in A3 AS. The total share of PAO, calculated as a sum of AccPAO and ActinoPAO, both for the activated sludge and the biofilm was higher than 40%. Its maximum share was reached during series A4, both in the activated sludge and the biofilm. It was also found that an increase of PAO in the activated sludge correlates linearly with a decrease of PAO in the biofilm (excluding the data from series A4; R^2^ = 0.93). The volume fraction of AccPAO in the biofilm was always higher than the volume fraction of ActinoPAO (Table 2).

**Table 2:** Domination within the group of *Accumulibacter phosphatis* (AccI-AccII) or PAO (AccPAO- ActinoPAO) or bacteria commonly present during the phosphorus removal process (PAO-GAO)

series	Competition between:					
	AccPAO I - AccPAO II		AccPAO - ActinoPAO		AccPAO - GAO	
	activated sludge	biofilm	activated sludge	biofilm	activated sludge	biofilm
**A1**	Acc II	Acc II	AccPAO	AccPAO	GAO	GAO
**A2**	Acc II	Acc II	ActinoPAO	AccPAO	PAO	PAO
**A3**	Acc II	Acc II	ActinoPAO	AccPAO	GAO	PAO
**A4**	Acc II	Acc II	ActinoPAO	AccPAO	PAO	PAO

In contrast, in the activated sludge, except for the first sample of the series, the PAO group is dominated by ActinoPAO. Candidatus “*Accumulibacter phosphatis*” was further researched to determine the dominance between clade I and II (none other clades where analysed). Both in the activated sludge and the biofilm, the dominant was *Accumulibacter phosphatis* clade II. Selected imeges from microscopic analyzes are shown in Fig. 4.

We also looked at the GAO group in the two fractions of the biomass. The volume fraction of GAO was the highest at the beginning of the experiment. Then the percentage abundance of GAO drastically diminished. The competition between AccPAO and GAO was exhibited as a presentation of the dominant one among the two groups (Table 2). Both the activated sludge and the biofilm show a surplus of GAO over AccPAO in series A1. From series A2 AccPAO, stays dominant in the activated sludge. In turn, in biofilm, it fluctuates indicating the dominance of PAO in the A2 and A4 series, and GAO in series A3.

The results of the Spearman rank correlation test showed that the ratio of AccPAO/GAO is correlated with the ammonium concentration in the effluent both in the activated sludge and the biofilm (Spearman determination coefficient of 1; *p*-value < 0.0001; α = 0.05). The proportion between AccPAO and ActinoPAO in the biofilm was found to correlate with the effluent concentration of total nitrogen and phosphorus with the same significance.

The qualitative analysis was made for known GAO representatives and some denitrifying bacteria at the end of the experiment. The sludge before the start of the experiment did not contain detectable amounts of *Defluviococcus* cluster 1, but there were a lot of highly dispersed *Defluviococcus* clusters 2. *D. vanus* was present only in the activated sludge. After more than 4 months of the IFAS-MBSBBR operation and a periodical change of the duration of the anaerobic phases, both biomasses revealed the presence of all the tested groups of *Defluviococcus,* where cluster 2 were the most abundant, then cluster 1, which had not been previously detected. *D. vanus* was visible but in small amounts. Surprisingly, among denitrifying bacteria, only the denitrifying cluster found previously in the methanol-utilising system was detected. There was no *Pseudomonas* and *Brachymonas*.

## Discussion

### Effect of technological parameters on bacterial community

Relevant technological factors in the nutrients removal by means of denitrifying dephosphatation that determine the effectiveness of the process are: 1) elimination of as much of the biodegradable organic compounds in the anaerobic phases as possible (their presence in wastewater in the anoxic zone would result in the course of denitrification using the carbon source present in wastewater rather than stored intracellularly by PAO in the anaerobic phase), 2) high-efficient nitrification ensuring a sufficiently high load of nitrates available as an ultimate electron acceptor in the respiration of DPAO in the anoxic conditions [11]. In order to ensure both technological requirements, it was necessary to optimise the length of the anaerobic phase (the phase without aeration) and the aerobic phases in the cycle, which was supposed to be 8 h. Brdjanovic et al. [18] showed that an extended aerobic phase might deteriorate the dephosphatation process due to excessive consumption of stored polymers. They did not find any explanation if excessive consumption is beneficial for PAO or GAO.

In the presented study the percentage share of the duration of the anaerobic phases with respect to the overall reaction time (without taking the sedimentation, decantation, and idle phase into account) is shown in Table 3. The efficiency of the removal of the organic compounds was higher than 95% (Table 1). It was noted that the extension of the phase without aeration resulted in a gradual increase in the efficiency of the biological phosphorus removal and an apparent reduction of phosphates in the effluent.

**Table 3:** Parameters used for DPAO enrichment in MBSBBR

Series	A1	A2	A3	A4
series duration, weeks	12.5	8	8	7
number of WW feeding	2	2	3	2
share of the anaerobic phases duration in the reaction time^a^, %	18	24	29	32

^a^ reaction time – the cycle duration excluding the sedimentation and decant phases

### PAO-GAO competition

In the anaerobic conditions, AccPAOs can take up organic substrates. They use intracellular polyP as the energy source and convert the organic compounds into polyhydroxyalkanoates (PHA). They may also use glycogen as a source of redox equivalents [19, 20]. In the following aerobic conditions, the PHA is used as a carbon and energy source to grow and recover the polyP and glycogen level. Glycogen accumulating organisms take up glycogen as the energy and reduction potential source to transform organic compounds. Thus, in the anaerobic conditions, PAOs and GAOs may compete for organic compounds [21]. Due to the similar physiology of PAO and GAO (organic substance uptake in anaerobic conditions, ability to respire in the presence of nitrate or nitrite), the overlap of ecological niches of the two groups is high, and therefore there should be a tendency for ruling out one of the two groups. The two groups coexist in most of the full scale enhanced biological phosphorus removal plants [22, 23] and laboratory systems [10, 15]. They are also found together in systems focused on denitrifying dephosphatation [15] and in the system presented in the study.

In our study, both types of biomass were dominated by GAO at the beginning of our study. Extending the share of the anaerobic phases resulted in the domination of AccPAO over GAO in the biofilm. When the wastewater feeding number had been switched from 2 to 3 (ensuring the same other technological parameters), GAO developed again to overgrow AccPAOs in the activated sludge. It seems the new conditions were more optimal for that group. The return to double feeding resulted in a reversion of the dominance and overgrowth of AccPAO. Variations in the populations of AccPAO and GAO may decrease the dephosphatation efficiency [22, 23], but in our case, the domination changes in series A3 and A4 did not affect the biological phosphorus removal which was the highest in the two series of experiment.

The most of the known factors that can influence and escalate the PAO-GAO competition (high temperature, carbon source, C:N:P ratio, oxygen concentration) were kept at a constant level and can be excluded from consideration. Furthermore, the age of the activated sludge was about 10 days, which should be the next beneficial factor for the growth of PAO. Such SRT is postulated to be beneficial for PAOs as GAO has a lower net biomass growth rate than PAO and dominate systems with long SRTs [24]. In fact, more of the time during the experiment, PAO dominated over GAO in the activated sludge.

### PAO community

The isolation of PAO is challenging, so identification of the leaders of the dephosphatation process requires the molecular methods to be applied. The most abundant bacterium in the EBPR systems is *Accumulibacter phosphatis*, currently regarded as the leading representative of PAO [4, 20] and also denitrifying PAO [4]. It belongs to *Rhodocyclus* group (Betaproteobacteria), and based on the *ppk* phylogenetic analysis, *Accumulibacter phosphatis* was divided into two types (I and II) within which several clades were distinguished [25]. Type I *Accumulibacter phosphatis* is able to denitrify from nitrate, while Type II from nitrite [4, 5].

In the study, a detailed view into the biocenosis relied on the detection of *Accumulibacter phosphatis* (AccPAO) as a whole, *Accumulibacter phosphatis* Type I (AccPAO I) and Type II (AccPAO II), and *Actinobacterial* PAO (ActinoPAO). The PAO group was initially dominated by *Accumulibacter phosphatis* Type II both in the biofilm and the activated sludge. The increasing differences between the activated sludge and the biofilm observed based on DGGE are also reflected in the domination among PAOs recognised by FISH. When the percentage of the anaerobic phases in IFAS-MBSBBR was higher than 18, the biofilm PAOs were still dominated by Candidatus *Accumulibacter phosphatis* Type II, but the activated sludge revealed a change to ActinoPAO and its further constant domination in that type of biomass. In the presented study, we observed from over a dozen to several dozens of AccPAO (or PAO), but the levels of the bacterial group’s percentage in the activated sludge and the biofilm were usually comparable. Apart from the fact that *Accumulibacter phosphatis* is the most commonly mentioned and investigated PAO species, very often an abundance of the PAO community is characterised only by counting of *Accumulibacter phosphatis* [4]. Meanwhile, more widely oriented research indicates that *Actinobacterial* PAO dominates over AccPAO in many WWTP [26] or possess the same abundance as *Accumulibacter phosphatis* [22, 27]. In our system, *Actinobacterial* PAO dominated the activated sludge for most of the research period and were present in the biofilm, although in the minority.

In the biofilm, where the bacteria grow inside a matrix of extracellular polymers which is denser than wastewater, the penetration time of compounds is longer. Thanks to this, the biofilm bacteria can be protected from external toxic compounds or short-term starvation [28]. AccPAO II was found to have a competitive advantage over AccPAO I due to distinct behaviour under phosphate limiting conditions [29]. As the phosphates concentration in the effluent was very low, often lower than 0.1 mg PO_4_-P/L, the bacteria were probably waiting for a new portion of P-rich wastewater. That could be a second factor that constituted a difference between community structures in the activated sludge and the biofilm. However, it has to be reminded that both Types of Acc were present in both biomass types. There is little known about the physiology and preferences of *Actinobacterial* PAO, especially in wastewater treatment systems. However, they play an essential role in phosphorus removal [30] and their representatives. Nevertheless, it would be beneficial to know how the percentage abundance relates to the net phosphorus uptake and the dephosphatation activity, especially concerning *Accumulibacter phosphatis*. It would also be important to answer the question of why the different species coexist and how they cooperate if they do.

### Denitrifying PAO

IFAS-MBSBBR was earlier used by [13] to obtain the organic carbon and phosphorus removal in the anaerobic phase and simultaneous nitrification-denitrification during the aerobic phases. It was found that the inner layer of the biofilm covering the carrier was colonised by bacteria capable of simultaneous denitrification-dephosphatation. In the presented study, the share of simultaneous denitrification-dephosphatation in overall dephosphatation was assessed according to biochemical tests [31] and presented as DPAO percentage abundance. The values assessed for the biofilm correlate linearly with the percentage abundance of AccPAO assessed by FISH (the coefficient of determination is 0.71). This observation exhibit the dominant position of *Accumulibacter phosphatis* in the biofilm and its share in the simultaneous denitrification and dephosphatation. There was no correlation between the assessed DPAO in the activated sludge and any other group of the analysed bacteria. Perhaps in the activated sludge, which is the part of biomass which is more exposed to changeable SBR-forced conditions, the bacterial community underwent different transformations or processes that do not allow to relate their abundance to denitrifying-dephosphatation activity simply. For example, reduced dephosphatation efficiency may be a consequence of using a glycolytic pathway by PAOs instead of P uptake [5, 20]. The PAO can reveal a typical PAO metabolism, a mixed PAO-GAO metabolism, or can adapt to the GAO metabolism [20]. When PolyP depletes (e.g. under low P/VFA conditions) PAO II reveals a much higher activity than PAO I showing a definite competitive advantage. Moreover, PAO Type II can use a mixed PAO-GAO metabolism in the anaerobic stage when the PolyP does not limit the uptake of VFA. Research using more sophisticated techniques such as FISH-MAR or sequencing is desirable to link the effectiveness of the denitrifying dephosphatation process to the species responsible for it, as well as confirm the findings obtained in the present research.

## Conclusions

- Wastewater treatment in IFAS-MBSBBR enables a synergic removal of nitrogen and phosphorus in one reactor based on the activated sludge and the biofilm biomass.
- High nutrient removal efficiencies were obtained via the denitrifying dephosphatation process. The higher efficiency was reached when IFAS-MBSBBR was operated with a 30% share of the duration of the anaerobic phases in the duration of the reaction phase. 91.1% of nitrogen and 98,8% of phosphorus was then removed from synthetic wastewater.
- The biocenosis of the biofilm and the activated sludge reveal different species patterns (according to DGGE) and domination of the EBPR community (FISH).
- Denitrifying PAO (DPAO) covered more than 90% of PAO in the biofilm and usually around 70% of PAO in the activated sludge.
- No correlation between anaerobic phase length and species share has been determined. However, when the IFAS-MBSBBR was operated with a higher percentage of anaerobic phases the activated sludge was dominated by ActinoPAO, while the biofilm by AccPAO.
- The extension of the duration of anaerobic phases caused an increase in the amount of denitrifying PAO (DPAO) in PAO.

## Methods

### Reactor configuration and performance

Denitrifying dephosphatation was investigated in the laboratory-scale IFAS-MBSBBR with an active volume of 28 L. The study consisted of four series (A1-A4), which differed in terms of the ratio of the anaerobic phases’ duration with relation to the overall reaction time in the cycle (Table 3). Each series was preceded by a 20-day period of biomass acclimation to the new conditions.

**Fig. 5:**
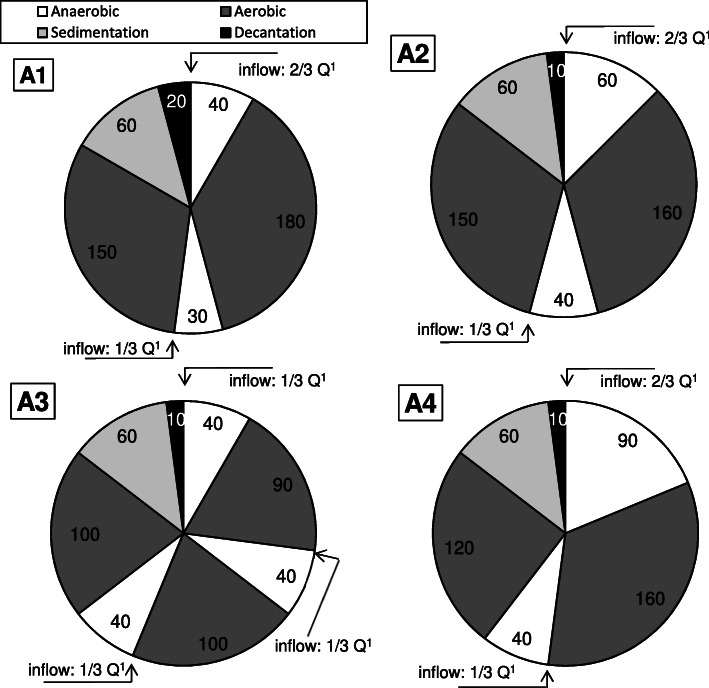
Cycle arrangement of the reactor in each series of the experiment (Q – the total amount of wastewater entering the reactor in the cycle, Q = 10 L/cycle)

In all the series, except series A3, two wastewater feedings in the cycle were used. In series A3 the number of feedings in the cycle was increased to three. In all the series the reactor was operated at three 8–hour cycles per day. The dissolved oxygen concentration was maintained at 6.0 mg O_2_/L. Throughout the whole study, the sludge retention time (SRT) was maintained at 10 d, and the concentration of the activated sludge averaged 2.89 ± 0.48 g/L. The arrangement of phases in the reactor’s cycles at each series, indicating the phase in which wastewater was supplied to the reactor, is shown in Fig. 5. The reactor performance was automated by using the DreamSpark Premium software (Microsoft). It was thoroughly mixed in both anaerobic and aerobic phases. The reactor was filled with EvU-Perl carriers (600 m^2^/m^3^ specific surface area and density 1.1 kg/L) up to 25% of the reactor’s active volume. The reactor was inoculated with activated sludge from a full biological nutrient removal WWTP. The experiment was preceded by a six-month start-up period due to the need for biofilm development on a moving bed. Synthetic wastewater, prepared once a day based on peptone, ammonium acetate, starch, glucose, Na_2_HPO_4_·12H_2_O, and KH_2_PO_4_, was supplied to the reactor in the anaerobic phases in a volume of 10 L per cycle (the decantation coefficient which describes the relationship between the volume of treated wastewater withdrawing during the 1 cycle and the maximum active volume of the reactor was 0.36).

The composition of raw wastewater remained the same in all the series (COD (chemical oxygen demand): 674 ± 58.6 mg O_2_/L, TN (total nitrogen): 64.9 ± 8.43 mg N/L, NH_4_-N (ammonium nitrogen): 38.5 ± 6.79 mg NH_4_-N/L; TP (total phosphorus): 9.88 ± 1.51 mg P/L; PO_4_-P (phosphate phosphorus): 8.12 ± 0.77 mg PO_4_-P/L, pH: 7.00–7.96). COD:N and COD:P ratio was 10.5 and 69.6, respectively. The reactor was operated in an air-conditioned room (temperature 18 °C). A detailed description of IFAS-MBSBBR can be found at Podedworna et al. [15].

The performance of the reactor was monitored by:

- the influent and effluent analysis for nitrogen, phosphorus, and organic compounds (COD, TKN, NH_4_-N, NO_2_-N, NO_3_-N, TP, PO_4_-P, pH, alkalinity) according to the APHA Standard Methods [32],
- the phosphorus uptake batch tests (PUBT) – conducted for the determination of the percent share DPAO in the total amount of PAO present in the activated sludge and the biofilm. The percentage share of DPAO in PAO was calculated as a ratio of the total phosphorus uptake under anoxic and aerobic conditions [31].

### Sample collection and fixation

Samples of the activated sludge and the biofilm were taken at the end of each series from the settled biomass at the idle phase. The biofilm was scratched from the carrier by sterile tools and rinsed by 1xPBS. Both the activated sludge and the biofilm used further for the denaturing gradient gel electrophoresis (DGGE) were frozen and kept at -20 °C until the sample analysis. Those biological materials used for the fluorescence in situ hybridisation (FISH) were fixed with 4% paraformaldehyde and stored at -20 °C.

### Denaturing gradient gel electrophoresis conditions and DNA bands extraction

Bacterial genomic DNA was extracted by glass beads (0.4–1.55 mm) beating enhanced by the addition of SDS. A clean-up DNA purification kit (A&A Biotechnology, Poland) was used as recommended by the manufacturer.

The extracted DNA was used as a template to amplify 16S rDNA through PCR with a pair of universal primers: 338F-GC, and 518R. PCR system: 5 μL of a template; 5 μL of MgCl_2_ (2 mM), 10 μL of 1xGoFlexi TAQ buffer, 1 μL of dNTPs (5 mM) of upstream and downstream primers, 0.25 μL of GoFlexi TAQ polymerase (1.5 U), and ddH2O was added to bring the volume to 30 mL. PCR procedures were as follows: denaturation at 95 °C for 15 min., 30 cycles of denaturation at 95 °C for 1 min., annealing at 53 °C for 1 min., and extension at 72 °C for 1 min.; the final extension at 72 °C for 10 min.; and termination at 4 °C. A no-template negative control was included for every PCR reaction. PCR products were analysed by the 1% agarose gel electrophoresis. Concentration and quality tests of DNA were performed by the NanoDrop 2000 spectrophotometer.

Electrophoretic separation of PCR products was made in the Dcode Universal Mutation Detection System (BioRad) in polyacrylamide gel (8%, 37:1 acrylamide-bisacrylamide, Fluka) with urea gradient of 35–60%. 5000 ng of PCR product was loaded onto the polyacrylamide gel and electrophoresed at a constant temperature (60 °C) by 14 h at 45 V in a 1 × TAE buffer (Tris, acetic acid, EDTA, pH = 8.0). Staining of the gel was made with SYBR Gold (1:10000, Invitrogen) in MiliQ water for 30 min. Following discolourisation in MiliQ water took for 40 min. The gel was visualised under UV light and recorded by Kodak 1D.

### Fluorescence in situ hybridisation

Identification of selected bacterial species or groups was carried out using FISH (fluorescence in situ hybridisation). EUB338, EUB338 II, and EUB338 III oligonucleotide rRNA (ribosomal ribonucleic acid)-targeted probes (Genomed, Poland) were used to detect all bacteria. The probes were mixed in a 1:1:1 ratio (EUBmix). PAO462, PAO651 and PAO846 were mixed in a 1:1:1 ratio to detect polyphosphate accumulating organisms (PAO) known as Candidatus “*Accumulibacter phosphatis*”(AccPAO); whereas Acc-I-444 or Acc-II-444 was used for detection of *Accumulibacter phosphatis* clade I (AccPAO I) or clade II (AccPAO II), respectively. GAOQ431 and GAOQ989 were mixed in a 1:1:1 ratio to detect glycogen accumulating organisms (GAO) known as Candidatus “*Competibacter phosphatis*”(GAO); Actino658 together with two competitors or Actino221 (as well with two competitors) for the detection of Actinobacterial PAO (ActinoPAO). For the detection of the *Defluviococcus* cluster I and cluster II, DEF998 and DEF1020 (with suggested helper probes) were used. *D. vanus* was detected with TFO_DF862. A few probes were selected at hoc for the detection of denitrifiers: Pdv1031 for *Pseudomonas denitrificans* and *Pseudomonas versutus*, OTU6–178 for *Brachymonas denitrificans* and DEN67 for the methanol-utilising denitrifying cluster. The probeBase database [33] was used to select proper probes and the optimal hybridisation conditions (hybridisation and wash buffer composition). In situ hybridisation was made according to the guidelines presented by Daims et al. [34]. Confocal laser scanning microscope (Olympus FluoView FV1000) equipped with an Ar-ion laser (488 nm) and two HeNeLasers (543 nm and 633 nm) was used for quantitative microscopic analyses. Area of fluorescent signal from the probes AccPAO, ActinoPAO, and GAO was quantified. The results of the quantification are presented as a percentage of the area of specific probe to all *Eubacteria* detectable by EUBmix. Quantification was based on randomly chosen images (30 images) recorded from each sample and probe. DAIME (digital image analysis in microbial ecology) program was used for the calculation of the specific probe percentage [35]. The qualitative analysis (together with a subjective visual estimation) was performed for clades of AccPAO, *Defluviococcus* organisms and selected denitrifiers. For that purpose, the fluorescence microscope MA300T (MOTIC) was used.

### Calculations and statistical analysis

The efficiency of a single process was calculated as a percentage of the removed concentration (total nitrogen or phosphates) or a parameter value (e.g. COD) concerning the concentration or value at the beginning of a cycle.

The standard error of the mean for the FISH quantitative analysis was calculated as the standard deviation divided by the square root of the number of images. The biomass volume in relation to all bacteria (EUBmix) was calculated as the area covered by the specific probe divided by the area covered by EUBmix.

The UPGMA clustering of DGGE bands was performed using GELCompare II (AppliedMaths) and XLSTAT (Addinsoft, France). Spearman’s rank correlation test was carried out with XLSTAT (Addinsoft, France).

## Supplementary information

**Additional file 1.** PCR-DGGE profiles of investigated samples. M – marker, 1 – A1 AS, 2 – A2 AS, 3 – A3 AS, 4 – A4 AS, 5 – A2 B, 6 – A3 B, 7 – A4 B, X – samples not discussed in the paper; AS - activated sludge; B - biofilm; A1 - A4 – series’ name (see: Table 3 for explanation).

## Data Availability

All data generated or analysed during this study are included in this published article.

## Abbreviations

AccI, AccII: *Accumulibacter phosphatis* Type I, *Accumulibacter phosphatis* Type II
AccPAO: Candidatus “*Accumulibacter phosphatis*”
ActinoPAO: Actinobacterial PAO
AS: Activated sludge
B: Biofilm
COD: Chemical oxygen demand
DGGE: Denaturing gradient gel electrophoresis
DNA: Deoxyribonucleic acid
DPAO: Denitrifying PAO
EBPR: Enhanced biological phosphorus removal
EDTA: Ethylenediaminetetraacetic acid
FISH: Fluorescent in situ hybridisation
FNA: Free nitrous acid
GAO: Glycogen accumulating organism
IFAS-MBSBBR: Integrated Fixed-Film Activated Sludge – Moving-Bed Sequencing Batch Biofilm Reactor
MAR: Microautoradiography
NH_4_-N: Ammonium nitrogen
NO_2_-N: Nitrite nitrogen
NO_3_-N: Nitrate nitrogen
PAO: Polyphosphate accumulating organism
PCR: Polymerase chain reaction
PHA: Polyhydroxyalkanoates
PO_4_-P: Phosphate phosphorus
PUBT: The phosphorus uptake batch tests
SRT: Sludge retention time
TKN: Total Kjeldahl nitrogen
TP: Total phosphorus
UPGMA: Unweighted pair group method with arithmetic mean
VFA: Volatile fatty acid
WW: Wastewater
WWTP: Wastewater treatment plant
