# Supplementary material for: Effect of anaerobic phases length on denitrifying dephosphatation biocenosis – a case study of IFAS-MBSBBR
Source: BMC Microbiol. 2020 Jul 24;20:222. doi: 10.1186/s12866-020-01896-3 (PMC7379833; doi:10.1186/s12866-020-01896-3)
Supplement: Supplementary file 1 — Additional file 1. PCR-DGGE profiles of investigated samples. M – marker, 1 – A1 AS, 2 – A2 AS, 3 – A3 AS, 4 – A4 AS, 5 – A2 B, 6 – A3 B, 7 – A4 B, X – samples not discussed in the paper; AS - activated sludge; B - biofilm; A1 - A4 – series’ name (see: Table 3 for explanation). [file 12866_2020_1896_MOESM1_ESM.docx]

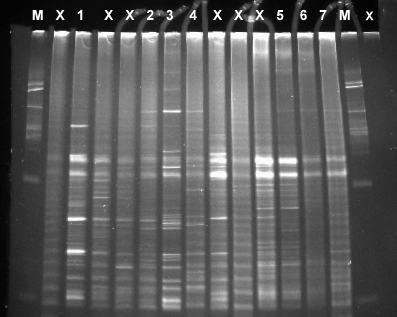


**Additional Figure.** PCR-DGGE profiles of investigated samples. M – marker, 1 – A1 AS, 2 – A2 AS, 3 – A3 AS, 4 – A4 AS, 5 – A2 B, 6 – A3 B, 7 – A4 B, X – samples not discussed in the paper; AS - activated sludge; B - biofilm; A1 - A4 – series’ name (see: Table 3 for explanation)
